# 300 Intraoperative Thresholds for Transfusion During Burn Surgery

**DOI:** 10.1093/jbcr/irad045.275

**Published:** 2023-08-29

**Authors:** Evan L Barrios

**Affiliations:** University of Florida, Gainesville, Florida

## Abstract

**Introduction:**

Burn injury is associated with significant morbidity. Deep partial thickness or full thickness burns require surgical excision and skin grafting. This process results in significant blood loss and frequently requires blood transfusions. There is scarcity in published literature addressing intraoperative blood transfusion requirements. The relationship between blood product volume and patient characteristics has not been clearly defined. The enhanced ability to predict blood transfusion requirements would allow for improved allocation of blood products and safer overall operations.

**Methods:**

This is a retrospective review conducted at a large academic burn center. Inclusion criteria are adult patients admitted between December 2019 and August 2020 with burns requiring surgical excision. Prisoners, children, and non-operative burns were excluded. Operative cases requiring blood product administration were compared to cases that did not require transfusion. Two-tailed student’s t-tests were conducted with p < 0.05 considered significant.

**Results:**

There were 103 total patients who underwent a total of 217 operations. Forty-eight percent (n=104) of cases required blood transfusion. As detailed in the table, the transfusion group had significantly increased percent total body surface area (TBSA) burn, along with longer hospital and ICU lengths of stay (LOS). Operatively, this group underwent more tissue excision (approximately 15% TBSA compared to 5%) with longer case lengths, coupled with higher estimated blood loss and a lower preoperative hemoglobin. Interestingly, the median time for the initiation of packed red blood cell transfusion was six minutes after incision.

**Conclusions:**

The average estimated blood loss in cases resulting in transfusion was 907±462mL (with a median of 800mL) compared to 467±398mL estimated blood loss in the non-transfused group. Expected blood loss along with parameters such as planned area of excision (which was approximately three times greater in transfused patients) and severity of burn injury can guide the surgeon in anticipating perioperative administration of blood products. Future directions include stratification of blood volume administration by %TBSA, operative time, LOS, and percent excision.

**Applicability of Research to Practice:**

The ability to predict blood product requirements preoperatively coupled with the incorporation of perioperative parameters can assist in the judicious and appropriate use of blood products during burn operations.

